# Acute Mesenteric Tuberculous Lymphadenitis: A Comparative Analysis of Twenty-one Cases

**DOI:** 10.7759/cureus.4454

**Published:** 2019-04-13

**Authors:** Asif Mehmood, Amna Ehsan, Maryam Mukhtar, Faisal Inayat, Waqas Ullah

**Affiliations:** 1 Internal Medicine, Geisinger Medical Center, Danville, USA; 2 Internal Medicine, Fauji Foundation Hospital, Rawalpindi, PAK; 3 Internal Medicine, Allama Iqbal Medical College, Lahore, PAK; 4 Internal Medicine, Abington Hospital-Jefferson Health, Abington, USA

**Keywords:** tuberculosis, tuberculous lymphadenitis, mesenteric lymphadenitis

## Abstract

Tuberculosis (TB) remains a major public health concern. Atypical extrapulmonary presentations of this infection may significantly delay its diagnosis and management. Tuberculous lymphadenitis (TL) is an extrapulmonary manifestation of a Mycobacterium tuberculosis infection. It is characterized by necrotizing mycobacterial infection of the lymph nodes. The clinical presentation of this disease ranges from fever and malaise to cervical lymphadenopathy and fistula formation. Herein, we present a comprehensive review of the previously reported cases of mesenteric lymphadenitis. The purpose of this study is to acquaint physicians to identify this disease in a timely manner. Furthermore, this review also highlights the rare presentation and management of TL.

## Introduction and background

Tuberculosis (TB) is one of the oldest diseases known to affect humans. It has been described in Chinese texts from as long as 2,300 years ago with the evidence of Pott’s disease noted in Egyptian mummies [1-2]. Mycobacterium tuberculosis deoxyribonucleic acid (DNA) has also been recovered in Peruvian mummies. Extrapulmonary TB most commonly presents in the form of tuberculous lymphadenitis (TL), which is a local manifestation of this disease [3]. Individuals may present with malaise, low-grade fever, weight loss, cervical lymphadenopathy or fistula formation. Diagnosis can be established using fine-needle aspiration cytology (FNAC) and a core biopsy. Pathologic examination of the biopsy specimens demonstrates granulomatous features typical of TB. We present a comprehensive literature review of previously reported cases of mesenteric lymphadenitis as an extrapulmonary manifestation of TB.

## Review

TB is one of the biggest health challenges in the world. The risk of this infection is greater in crowded populations and it is particularly prevalent in the lower socioeconomic setting. It is the most common diagnosis for cervical lymph node enlargement in developing countries [4]. According to one estimate, approximately 2.2 million people acquire TB every year in India, out of which 0.87 million cases are infective and are responsible for infecting further individuals and spread of the bacterium [5].

TL is the most common manifestation of extrapulmonary TB in human immunodeficiency virus (HIV)-seronegative and HIV-positive individuals. Other common extrapulmonary manifestations of TB include symptoms related to pleura/pericardium, genitourinary system, Pott’s disease, etc. TL makes up about 35% of extrapulmonary TB. Mohapatra et al. showed that it has a female predominance and also has a higher prevalence in HIV-infected individuals [6]. TL is a local manifestation of a systemic disease that can occur as a primary disease, reactivation, or direct extension from a contiguous disease focus. It typically presents as scrofula-cervical lymphadenopathy, fistula formation, and typical constitutional symptoms, especially low-grade fevers and weight loss. Travel history is important for the diagnosis of TL as many individuals travel to and from endemic areas of TB and some remain asymptomatic.

We conducted an extensive literature search that retrieved 21 articles. After reading the titles for relevance, 18 articles were excluded, one article did not have enough data, and a total of two articles were selected. One out of these two selected articles reported 20 patients. Therefore, a total of 21 patients with TL were included in this review. All patients of TL were females with a mean age of 43 years (range: 23-69 years). Abdominal distension was the most common presenting feature (57%, n = 12), while 52% of patients presented with abdominal pain. Fever was present in 19% of individuals (four cases) while dyspnea was present in 9% individuals (two cases). Other presenting complaints included constipation, menorrhagia, and peritonitis (one case each). About 52% of patients had primary peritoneal carcinoma (PPC) along with tuberculosis, while ovarian tumor was present in two cases each. Only one case reported ulcerative colitis as a comorbid condition. All individuals with TL had a computed tomography (CT) scan done in their diagnostic work-up (100%, n = 21).

All patients with TL were treated surgically, in which peritoneal biopsy was most commonly used (47%, n = 10). Laparotomy was performed in 38% of patients (n = 8) while 19% of individuals had bilateral salpingo-oophorectomy and omental biopsy was done (four cases each). Only 9% cases had a total abdominal hysterectomy performed during surgery (two cases each) while omentectomy, mesenteric biopsy, unilateral salpingo-oophorectomy, adhesiolysis and biopsy, salpingectomy, mass excision, pulmonary nodule resection, and lobectomy were done in one case each. Out of the 21 cases, 95% of patients had a satisfactory outcome with relapse of TB reported in only one individual [7-8]. The characteristics of these patients are shown in Table 1.

**Table 1:** Characteristics of previously reported cases of mesenteric tuberculous lymphadenitis TB: Tuberculosis; CT: Computed tomography; PPC: Primary peritoneal carcinoma; TAH: Total abdominal hysterectomy; BSO: Bilateral salpingoophorectomy.

S. No	Author	Age/Sex	Presentation	Comorbids	Investigations	Treatment	Outcome
1	Kraft A et al. [7]	46/F	N/A	Ulcerative Colitis	CT scan	Resection of pulmonary nodule, lobectomy	Relapse of TB
2	Choi Hun et al. [8]	46/F	Abdominal pain	Primary peritoneal carcinoma (PPC)	CT scan	Total abdominal hysterectomy (TAH), Bilateral salpingoophorectomy (BSO), Omentectomy, Laparotomy	Satisfactory
3	Choi Hun et al. [8]	64/F	Abdominal pain	PPC	CT scan	Peritoneal biopsy	Satisfactory
4	Choi Hun et al. [8]	28/F	Abdominal pain, Abdominal distention, Fever	PPC	CT scan	Peritoneal biopsy	Satisfactory
5	Choi Hun et al. [8]	40/F	Abdominal pain, Fever, Constipation	N/A	CT scan	Peritoneal biopsy	Satisfactory
6	Choi Hun et al. [8]	33/F	Abdominal distension	PPC	CT scan	Peritoneal biopsy	Satisfactory
7	Choi Hun et al. [8]	26/F	Abdominal pain, dyspnea	N/A	CT scan	Laparotomy, Mesentery and omental biopsy	Satisfactory
8	Choi Hun et al. [8]	66/F	Abdominal distension	N/A	CT scan	Peritoneal biopsy	Satisfactory
9	Choi Hun et al. [8]	23/F	Menorrhagia	Ovarian tumor	CT scan	Peritoneal biopsy, Unilateral salpingoophorectomy, Laparotomy	Satisfactory
10	Choi Hun et al. [8]	69/F	Abdominal distension, Dyspnea	PPC	CT scan	BSO, Peritoneal biopsy, Laparotomy	Satisfactory
11	Choi Hun et al. [8]	69/F	Abdominal distension	PPC	CT scan	Peritoneal biopsy	Satisfactory
12	Choi Hun et al. [8]	25/F	Abdominal pain	N/A	CT scan	Peritoneal biopsy	Satisfactory
13	Choi Hun et al. [8]	62/F	Abdominal pain, Abdominal distension	PPC	CT scan	BSO, Omental biopsy, Laparotomy	Satisfactory
14	Choi Hun et al. [8]	38/F	Abdominal distension	N/A	CT scan	Peritoneal biopsy	Satisfactory
15	Choi Hun et al. [8]	23/F	Abdominal distension, Abdominal pain	N/A	CT scan	Omental biopsy	Satisfactory
16	Choi Hun et al. [8]	48/F	Abdominal pain, Abdominal distension	PPC	CT scan	Peritoneal biopsy	Satisfactory
17	Choi Hun et al. [8]	41/F	Abdominal distension	PPC	CT scan	TAH, BSO, Laparotomy	Satisfactory
18	Choi Hun et al. [8]	53/F	Fever, Peritonitis	N/A	CT scan	Laparotomy, adhesiolysis, biopsy	Satisfactory
19	Choi Hun et al. [8]	36/F	Abdominal distension, Abdominal pain, Fever	Ovarian cancer	CT scan	Peritoneal biopsy	Satisfactory
20	Choi Hun et al. [8]	36/F	Abdominal distension	PPC	CT scan	Omental biopsy	Satisfactory
21	Choi Hun et al. [8]	38/F	Abdominal pain, Abdominal distension	PPC	CT scan	Laparotomy, Salpingectomy, mass excision	Satisfactory

Although there is no age limit for this disease, there are cases of TL seen in infants as well as those above 60 years of age. A study performed in Chandigarh revealed that TB is more commonly seen in younger age groups (10-30 years) [3]. Another study revealed that the highest incidence of TL in the United Kingdom and United States is 25-50 year age-group [9-10]. The higher number of cases have been reported in females with the male-to-female ratio of 1:1.2 in a study performed by Dandapat et al. and 1:1.3 in a study by Subrahmanyam [4,11]. It is proposed that probably women are more conscious of their physical appearance and have a lower nutritional status relative to males in a male-dominated society [1].

There are multiple modes of presentation of the disease, the most common of which are the systemic symptoms of malaise and weight loss [3]. A study conducted by Patel et al. noted weight loss in about 77% and fever in 73% of the cases [12]. While in another study by Dandapat et al., it was observed that weight loss was seen in 85% and fever in 40% of the patients [4]. The primary system affected by TB is the bronchopulmonary apparatus, with the head and neck commonly being secondary; therefore, symptoms related to these organs are also noted when they're involved [6]. An abscess or a discharging sinus tract may be another manifestation of disease in patients with TL [13].

The diagnosis of TB can prove to be tricky in cases with extrapulmonary involvement, but three features can help ascertain the diagnosis of TL, they are a multiplicity, matting, and caseation [14]. FNAC is a very popular diagnostic tool for the assessment of cervical swellings. In developing countries where TB is more common than other granulomatous conditions, the presence of granulomatous features on FNAC is highly suggestive of TB [15]. The diagnosis is further strengthened by observing the Mantoux test and erythrocyte sedimentation rate (ESR), thus eliminating the need for excisional biopsy in most of these patients [3].

Before the advent of chemotherapy, the major mode of treatment of TL was surgical excision of the involved lymph nodes [16]. When chemotherapy for TB was introduced in the 1950s, anti-TB chemotherapy for 12 to 24 months was given after excision of all the involved lymph nodes as an effective means of treatment [17]. A study by Cambell et al. revealed that a daily six-month chemotherapy regimen has proven to be successful for TL [18]. Studies by Patel et al. and Yuen et al. also confirmed that all the patients could be successfully treated with a short course of six-month chemotherapy with a very rare need for surgical intervention [12,19].

## Conclusions

Extrapulmonary TB, although not as common as pulmonary TB, does exist in the United States so, an inquiry about the country of origin and travel history in a patient with isolated cervical or diffuse lymphadenopathy could help narrow down the differential diagnoses. Endoscopic ultrasound, fine needle aspiration, and fine needle biopsy are useful tools for the diagnosis of TL, especially if the abdominal lymph nodes are the most easily accessible. The chemotherapeutic approach is effective in most patients and surgical intervention can be avoided.
